# Atlanta Academy of Medicine

**Published:** 1876-06

**Authors:** R. W. Westmoreland

**Affiliations:** Reporter


### Atlanta Academy of Medicine.

R. W. WESTMORELAND, M.D., Reporter.

Atlanta, April 14, 1876.

Academy met, the President, Dr. Miller, in the chair.

Dr. R. W. Westmoreland reported a case, which was supposed to be œzena, but which proved to be a cherry-seed lodged in the posterior nares. This, from the patient's account, must have remained fixed for a number of years.

Dr. W. F. Westmoreland reported the case of one of the subjects of the ham poisoning that happened in the city not long since. The patient, aged seventy-four years, had been sick for two or three weeks after eating the ham, when he was taken with symptoms similar to those of intestinal obstruction. Injection of water was given, and about a gallon forced in the bowels without effecting an action, or without any return of the water. A day or so afterward another gallon was injected, followed by the discharge of some fecal matter and about a quart of the injection. On the next day another gallon was injected. Although the patient made efforts to defecate, he failed, and after lingering a short while, died. Post mortem showed the ascending colon to be enormously distended, measuring about ten inches in diameter. Found several patches of ulceration near ilio-cœcal valve.

Dr. John M. Johnson mentioned cases of poisoning by eating meats confined in tin vessels.

Dr. Miller considered that in the case of Dr. W. F. Westmoreland, local and not general paralysis, with irregular peristaltic action, was the cause of the difficulty.

Dr. J. P. Logan mentioned the strange occurrence of waiters at the hotel dropping down in epileptic fits. This occurred with several waiters the same day. Did not pretend to account for it, but mentioned it as a novel coincident.

Dr. John M. Johnson reported a case of impotence in a man who formerly was addicted to masturbation, and asked opinion of members in reference to it.

Dr. Bragg explained the inquiry made to Dr. Johnson as to the constriction of the meatus, by saying that he once saw a case of impotence cured by opening up the meatus.

Dr. W. F. Westmoreland thought stricture often the cause of such affections, and mentioned cases where the impotence resulted from and was cured by removal of stricture.

Dr. V. H. Taliaferro remarked that his experience was in corroboration of Drs. Westmoreland and Bragg, and thought that excessive chronic irritation of the urethra, of any kind, might cause impotency. He mentioned cases where irritation along the canal, without any stricture, was accompanied by impotency, which was relieved by curing the irritation.

Dr. R. W. Westmoreland reported an operation at ankle, where the joint was so fractured and lacerated that it had been decided to amputate the leg; but on further investigation it was thought proper to preserve the length of the limb by Pirigoff's operation. This he accordingly did, without dividing the posterior tibial artery, thereby preserving the circulation in the heel portion of the stump. At this time—six days after operation—the patient is doing well, with every prospect of a successful operation and speedy recovery.

Dr. Calhoun reported two cases of enucleation of eye-ball. The first patient æt. fifty-four years; North Georgia; left eye affected. The eye began to pain him in August, 1875; the ball gradually increasing in size, and the pain daily increasing in intensity, until it had begun to protrude laterally between the lids, the vision being entirely lost, and the pain so intense that he was robbed of all sleep and rest. The pupil was widely dilated, with a bright greenish reflex; the lens was cataractous and distinctly visible up to the periphery. Towards the external and internal canthi, from which the tumor was found protruding, the sclera was very thin, displaying the dark pigment of the choroid underneath. He was taking daily large quantities of morphine, but with little relief. There was nothing of a cancerous diathesis, either hereditary or acquired, found in the history of the patient. In April, 1876, enucleation was made in the usual way; the arteria centralis retinæ was found very much enlarged, and the parts posterior to the ball very much congested, and an unusually free hemorrhage followed the operation, the surrounding tissues becoming fully infiltrated with the blood. This, however, was absorbed in a few days, the patient recovering rapidly; all pain ceasing immediately after the removal of the ball. No adverse symptoms presenting, he was dismissed at the expiration of ten days, cured. He has since been heard from, and is perfectly well.

Patient No. 2, æt. fifty six; Atlanta, Georgia; artist. When two years old, the patient, in playing, received a penetrating cut from a knife, the blade entering the interior of the eye at the lower corneo-sclerotic junction, wounding the ciliary bodies, and resulting in inflammation and entire loss of vision, with atrophy of the ball. In March, 1874, the patient first presented himself, saying that the vision of his right eye was rapidly failing, without pain. Upon examination, all the symptoms of progressive sympathetic inflammation were found. He was advised to have the left eye enucleated immediately, the dangers of losing the vision in the right eye being clearly shown and explained to him. However, he deferred the operation, and was not again seen until May, 1876, when it was found that all the vision that existed at the previous examination, one year ago, was lost, through the progress of the sympathetic inflammation, only a good perception of light remaining. The pain had not only increased in the left, or injured eye, but was now growing severe in the right eye. With the view of retaining this small remnant of light, and of being relieved of the constant pain, the left (injured) eye-ball was now enucleated, from which the patient made a rapid recovery, the pain ceasing, and the formerly still diminishing perception of light remaining as it was before the operation.

Worthy of special note in this case, is the length of time intervening between the injury and the appearance of the sympathetic inflammation, viz, fifty-two years.

My experience is, that injuries in the region of the ciliary bodies, resulting in loss of vision, sooner or later bring about sympathetic inflammation in the sound eye, sometimes after the lapse of a great number of years, as in the above case. Therefore it is advisable to remove the injured and sightless organ, with the view of preserving intact the uninjured one.

---

Atlanta, April 11th, 1876.

Meeting called to order by the President, Dr. Miller. Report of cases.

Dr. Calhoun reported a case of partial deafness in a lady aged twenty-six years, who, when fifteen years old, had scarlet fever, causing some lesion of the middle and internal ear. Latterly she has been quite deaf, and he had no hope of benefiting her in this respect. One strange feature of the case, that he desired to call attention to, is that although quite deaf, she can carry on a perfect conversation by noting the movement of the lips. At each catamenia, however, which has been irregular since her 15th year, she loses this faculty entirely, and seems to be utterly incapable of recognizing this means of conversing. He remarked that there were schools in Germany where the deaf were taught to understand by movement of the lips what was said. Could not account for the periodic aberration that destroyed this power of visual interpretation.

Dr. J. T. Johnson mentioned a report he had noticed of some of the schools referred to by Dr. C.; remarked that here the apter scholars advanced to the extent of articulating very plainly.

Dr. Calhoun supplemented by saying that he has often seen these "speaking classes," but that they fail to speak so as to be well understood.

Dr. Miller was of opinion that in the case of Dr. Calhoun the failure was not in vision, but in inability of the mind to receive the impression. Mentioned a case of imperfect articulation, attributable, as he supposed, to mental defect—a failure of the mind to form the idea necessary to speak of some certain thing.

Dr. Connally explained an inquiry as to existence of paralysis in Dr. Miller's case by mentioning a case of a young man in the city, who, being in the habit of talking very fast, breaks entirely down, and cannot say a word.

Dr. Todd mentioned the case of his preceptor, who on account of paralysis of tongue, was unable to speak at times.

Dr. Connally reported a case of sub-acute cystitis, excited by horse-back riding, treated by purgatives, followed by full doses of wine of ergot, and small doses of iron, according to the suggestion of Bumstead. Symptoms passed off, by constant use of the remedies, in four or five days. In reply to Dr. Taliaferro, said the desire to urinate was very frequent.

Dr. Taliaferro inquired if members were aware of any aphrodisiac qualities belonging to ergot. He regarded it as being very decided in that tendency. The remedy had been given in two cases, where it had this effect—headache and enlarged testicle.

Dr. W. F. Westmoreland reported case of excessive irritation of urethra of a lady aged 25 or 30 years. He, the last of a number, was called in, and found slight stricture just behind the meatus. Used the divulsor without much benefit following. Cauterized with thirty grain solution of silver, which caused some hemorrhage. Attempted to dilate with sponge tent, but, pain being too great, anæsthetized and dilated with divulsor by making slight incisions of the meatus. The urethra seemed to be in a granulated condition.

Academy adjourned.

---

ATLANTA, May 2, 1876.

Meeting called to order by the President, Dr. Miller.

Dr. R. W. Westmoreland reported the case of a patient affected with epileptic fits. Thirteen years ago the patient had an attack of scarlet fever, and a catarrh of the ear was the result. Three years after, the catarrh still existing, he had his first convulsion. He desired the opinions of members as to the cause and the proper mode of treatment in the case.

Dr. J. G. Westmoreland thought catarrh of the ear was the cause of the trouble, and if that was removed the convulsions would probably cease.

Dr. Calhoun thought that caries of bones of the ear was the origin of the trouble, and to cure that, might clear up the case, and perhaps benefit the patient.

Dr. Miller inquired if caries of teeth would not cause epileptic fits; thought that removal of cause would not cure the fits every time.

Dr. Connally inquired if members had seen any typhoid fever; has a case under treatment himself.

Dr. Taliaferro remarked that he had not seen any, but thought that as it always depended on local causes, we were liable to have it at any time.

Dr. Connally could not think that there was any local cause producing it in the case reported.

Dr. J. G. Westmoreland believed that atmospheric influence existed, independent of local cause.

Dr. Jno. Thad. Johnson agreed with Dr. W., and did not believe in the special theories of enthusiastic doctors of the day.

Dr. Taliaferro said that it would be found that this atmospheric influence always depended on local causes.

Dr. Jno. M. Johnson believed that some particular poison, either from bad air, food or water, was the origin of the disease. Mentioned the case of a family who ate of wheat which had become damp and mouldy from remaining in the field too long uncut, and all of them had typhoid fever. Mentioned typical cases of fever from vitiated food, water, etc. Thought that a particular pathological condition had as much to do with contracting the disease as anything else, and that the fever itself was contagious in certain of these conditions.

Dr. J. G. Westmoreland remarked that his experience was that typhoid and remittent fever did not go together; that they were dependent on different conditions, and were found to exist in different localities.

Dr. J. M. Johnson did not agree with Dr. W., but mentioned cases where they existed in the same locality.

Dr. Taliaferro thought a peculiar diathesis had something to do with contracting typhoid fever. Spoke of bilious fever, so called by physicians in the locality, as resembling typhoid fever. He treated it pretty much as he would bilious fever, with good results.

Dr. Connally mentioned the fact that physicians in Southwest Georgia were of the opinion that typhoid fever never existed in that section.

Dr. Miller coincided with Dr. J. T. Johnson that typhoid fever does not always depend on local causes; thinks it does at times. Believes with Dr. J. M. Johnson that it is contagious, and cited typical cases. Thought that typhoid and remittent fever might exist in the same locality.

Discussion terminating here, on motion, the Academy adjourned.

---

J. S. TODD, M.D., Reporter.

---

Atlanta, May 9, 1876.

Academy met, the President, Dr. Miller, presiding.

The following instructive and interesting case, reported by J. W. Oslin, of West Point, was ordered printed. The case illustrates the great importance of discrimination in taking the statements of patients for truth, and the necessity of instrumental examination before a diagnosis should be announced, in such diseases as stricture, fistulous openings, stone in the bladder, etc. In this case, until the probe was used, all was obscurity, and gave rise to discussions as to the probability of hernia (for he first located the tumor in labia majora), cystic tumor, hæmatocele, fistula in ano, and caries of pelvic bones:

Mrs. ———, age twenty-nine years; corpulent; bilious temperament. Menses appeared at the age of eleven years; monthly flow very free. Was married at the age of twenty. Since marriage she has taken on flesh very rapidly. She has suffered constantly with sick headache ever since the menses were established. There has been considerable disturbance of the liver for twelve or fifteen years past; digestion often very bad; the liver is frequently enlarged and tender; she frequently vomits large quantities of bilious matter.

First confinement March, 1871; duration twenty hours; the pains very violent from the first—the patient says almost one continual pain. The child was very large and well-developed, and lived but a few moments after delivery. Three days after delivery patient had a rigor. At this date I was called to see the patient for the first time. Found her with high fever; pulse 130; skin hot and dry; bowels much swollen and distended; the womb very large and tender to the touch. The indications were that the patient was attacked with puerperal fever. Under vigorous and prompt treatment, fever yielded in about ten days; patient able to sit up a little in six weeks. Three months after confinement, was called to see the patient again. Womb still enlarged and tender; suffering with continual pain in left iliac region; discharge from vagina free and constant. Made examination with speculum; found os and cervix much engorged, and a cicatrix of cervix about three-quarters of an inch in length. There was extensive abrasion of mouth and neck of womb. Treated case successfully, and dismissed patient.

Was called to see patient again in May, 1872, and again in December, 1873, at which times patient was delivered, without difficulty, of fine, well-developed children. Recovery in both instances good. General health as usual, until some six or eight months after third confinement—say July, 1874. About this time a tumor, soft and yielding, made its appearance in lower third of the lesser labia, on left side. The tumor was a painless one, except that occasionally sharp pricking pains were present; they were of very short duration. When patient was recumbent tumor disappeared, but was always present when in the erect posture, or at stool, or in a stooping position. The act of evacuating the bowels or of micturition always enlarged tumor. About five months after appearance of tumor, patient had a violent attack of what was said to be bilious colic. (Did not treat the case.) About one or two months from this time, patient was violently attacked with severe pain in bowels, vulva, womb, and region of tumor. Was called hurriedly to see her, and found great difficulty in diagnosing case. In eight or ten hours after seeing patient, discovered that the tumor was the centre of excitement. The adjacent tissue became very much inflamed and indurated, and the result was a large abscess, maturing and rupturing in about ten days. The next active abscess about six months from rupture of first. From that time until the present they have been forming at irregular periods; the tendency has been for the interval to shorten. The discharge from abscess has had all the characteristics of pus.

Most of the foregoing information I have derived from the patient. Was with her in confinements, but never treated the tumor.

The last of February, '76, was called to see patient. Found her suffering with an abscess, that formed about first of month. It had been discharging continuously, and for the first time the secretion was very offensive, and of dark color, with occasional small clots of blood. A speculum examination revealed the fact that the tumor was open; yet some obstruction prevented a free exit of matter. Finding the tumor large and fluctuating, I made a free incision with bistoury. Discharge very free, affording instant relief. Several abscesses have formed since that time. A very large one formed in the early part of March, from the effects of tincture of iodine having been injected into the cavity of tumor. Have made several free incisions; they heal up rapidly. The tumor always seems to contain pus. The patient informs me that she can easily rupture it with finger nail. It never discharges except when active inflammation has occurred, unless patient ruptures with finger. Says she can make it discharge at pleasure. I have neglected to state that after the first or second abscess had formed, that the tumor was no longer present when patient was standing or stooping.

Patient has suffered with internal hemorrhoids for several years. The ulceration is very extensive and very high up the rectum. Considerable loss of blood at almost every stool during the past year; pain and a sense of weight always present in rectum. Commenced treating hemorrhoids first of March; no hemorrhage since. The pain, however, has not abated in the least; continual aching in left side of pelvis; sharp, lancinating pain, radiating from tumor towards rectum and in bowels. Patient has been subject to constipation for twenty years.

The following letter to Dr. Taliaferro, in answer to certain queries, explains itself. Dr. T. particularly requested that a probe be used, and that he examine carefully himself, and not trust to what the patient said:

West Point, Ga., May 9, 1876.

My Dear Sir—Yours of 4th inst. received and contents carefully noted, and in reply I will proceed to answer your inquiries to the best of my ability: 1st. The seat of the abscess is evidently in the labia minora, but the labia majora has been more or less involved in several instances. 2d. A speculum was not necessary to bring the tumor in view. The first time I discovered, or rather examined, the tumor, I was making speculum examination of the womb, and on withdrawing the instrument I engaged the tumor between the blades of the instrument, pressing back labia majora, thus bringing it in full view. Subsequent examinations without instrument. 3d. On one occasion I opened abscess after it had fully matured, and caused the pus to run out. Then injected iodine into cavity. Can't say whether or not this cavity is a sac in labia minora or a cystic cavity in tumor. 4th. The tumor, or abscess, has certainly been discharging very constantly since the first abscess formed. The tumor was in existence several months before pus was discharged. There has certainly been an opening most of the time. The patient causes the tumor to discharge by pressing with finger on the adjacent tissue. When there is uneasiness in the parts, the patient forces a discharge by pressure. There is no scab ever present. The opening seems to close up, and seldom, as a general rule, discharges unless there is pressure upon the adjacent parts. The secretions collecting, there is pain or uneasiness in the tumor. It is then that the patient relieves herself by pressure. This she has been in the habit of doing every twenty-four hours. The tumor did not discharge for seven or eight days recently, that is, from yesterday week to Saturday. The discharge was very slight.

May 8. I visited the patient this morning, and placing her in proper position, proceeded to probe the sinus. The orifice has reduced in size of late. This A.M. it was so small that I discovered it with great difficulty. I with some difficulty passed the probe to the rectum, but could not make it enter. The point of contact is about an inch above sphincter ani. After satisfying myself of the direction of the sinus, I brought down the hemorrhoidal tumors with great difficulty, and ligated the two largest. I am of the opinion that the sinus has been gradually diminishing to the present time. I reason thus: A complete fistula had been established, causing the offensive odor. The application of caustic to the hemorrhoidal tumors came in contact with the sinus, or opening; the bowels, from the use of opium, were kept constipated a few days, thus favoring the closing of the opening in rectum, leaving a blind sinus from the point where pus has been discharging, to rectum. I shall await the result of ligating hemorrhoidal tumor, and if the tumor still discharges, I shall then divide sphincter ani, extending incision to tumor. I am of the opinion that the opening (if there has been one) in rectum is very high up. The direction of probe was upward, so as to come in contact with rectum about one and a quarter or one and a half inch above sphincter. I could have easily perforated by pressing probe on finger, but declined doing so.

I have answered your inquiries and given you some additional information. Many thanks for your kindness.

Respectfully, J. W. Oslin.

Dr. Taliaferro—The questions propounded to Dr. Oslin were with the view of clearing up some points in his case not made altogether clear by his written statement. The tumor is stated to have been in the labia minora, and during some months subsequent to its appearance was soft and elastic, without any evidence of inflammation, receding and disappearing in the recumbent posture, and reappearing when erect or sitting. Such a history, locating the tumor in the labia majora, would have strongly led to the supposition of labial hernia. Not so with the tumor in the nymphæ, for here we would not have a hernia without some anatomical anomaly. It appears from the Doctor's statement that the history of the tumor, up to the time of its inflammatory character, was obtained solely from the patient. This fact, taken in connection with the improbable, if not impossible, nature of the tumor as described by patient, renders the entire early history of the case unreliable, and hence valueless as a guide to correct diagnosis. These facts, which have been gathered by medical observation, alone can bring us to reliable and correct conclusions. Such facts date from the inflammatory character of the tumor. These, with the subsequent history, point very clearly to recto-labial fistula. The opening in the rectum here (doubtless its usual site, just above the sphincter) has terminated in the labia minora, instead of, as usual, near the verge of the anus. Should further exploration of the rectum be necessary for the ligation of tumors, or for diagnosis, I would suggest forcible dilatation and rupture of the sphincter ani muscle, instead of incision, as proposed by Dr. Oslin. This I would accomplish by etherizing the patient and inserting into the rectum the two thumbs, back to back; seizing the nates on either side, and making gradual traction until the thumbs touch the tuber-ischii. The paralysis following this remains for two or three weeks, but is never followed by permanent loss of power, which is the result sometimes of division. Thorough exploration may then be made by the touch, and with Sims' speculum by the sight, after this is accomplished. This operation would have, too, the additional advantage of destroying for the time being sphincter action, thus removing the persistent obstacle to curative measures.

Dr. Miller prefers the term fistula-in-ano opening in labia minora.

Dr. Taliaferro desires to call attention to an error in the published proceedings of the Atlanta Academy of Medicine in the May number of the ATLANTA MEDICAL AND SURGICAL JOURNAL, in which he is made to say, in reporting a case of post partem hemorrhage: "The hemorrhage he attempted to check by introducing his hand into the uterus and manipulating with the other hand on the outside, but produced no contraction or control of hemorrhage." What he did say, and the impression he desired to convey, was exactly the reverse of this. So soon as the alarming hemorrhage, which almost immediately followed birth of the child, was detected, the hand was carried into the uterus to the fundus, and failing to observe the usual prompt response by contraction and closure of the uterine walls upon the hand, the other hand was made to clasp the uterus through the abdominal walls, and the bleeding uterine vessels between the hands compressed; the hemorrhage thus was *completely arrested*, though there was not sufficient contraction of the uterus to be felt by the hands and make solid its walls. The hands were thus kept for little over an hour, and the *hemorrhage controlled*, until sufficient contraction could be induced by ergot. Again, in same issue of JOURNAL, he is made to say, in reply to Dr. Todd, as to his experience with fluid extract of ergot and ergotine, "that it is unfavorable." He stated that his experience was unfavorable with ergot indiscriminately obtained, owing to its poor quality, or its impaired virtues by the mode of preparation, as so often occurs in the fluid extract. He has implicit faith in a pure article, and relies with confidence on its specific action upon the uterus in all cases and conditions requiring its use.

Dr. Miller thought Dr. Taliaferro was right in practice when he introduced his hand into the uterus, but wrong in theory, when he considered that such a procedure would check hemorrhage from the uterus, except acting as an irritant and causing contraction of the organ. The fist could not possibly be brought in contact with all the bleeding uterine sinuses.

Dr. Taliaferro contended that so long as the uterus was flaccid—not solidly contracted—there would be hemorrhage, unless mechanically stopped. He knows this womb was flaccid, and that pressure, as above described, did arrest it. The hemorrhage would certainly have continued had his hand been removed. He, of course, would not tampon the vagina in post-partem hemorrhage, but would tampon the womb, when the hand could be applied as the tampon to the mouths of the bleeding vessels, as in this case.

Dr. Simpson reported three cases of what, for a better name, he designated scarlatina. One case had fever, diarrhœa, vomiting, and sore throat, but no eruption. In two there was an eruption, but no sore throat. The eruptions were papular; had quite a rough feel to the hand when passed over them. The eruption was confined to neck and joints. The cases in which there was an eruption were followed by desquamation. In no case was the tongue typical. All were slight. The urine was not examined for albumen. Hydropic effusions were not observed as sequelæ. All the cases were in the same family.

Dr. J. G. Westmoreland had seen during the past year several cases of same description. The eruption was papular, otherwise they did not differ from scarlet fever. He has given the affection no name.

Dr. Calhoun related several cases as illustrative of the hereditary tendency of eye affections. In a boy of thirteen, complaining of gradually increasing blindness for the last three months, the ophthalmoscope revealed opacity of posterior cortical substance of the lens (post-cortical cataract). Saw the mother of boy this evening; she has cataract of same character, only it is mature. Her age is thirty-five. It began on her when twenty-two years old. Her mother had same disease at her age. Her grand-mother—the boy's great grand-mother—was similarly affected in old age. There is in the Blind Academy, at Macon, in this State, a family known as the "blind family." The father has been blind since the age of four. He had nine children, six of whom were affected with the same character of cataract, which began in each at the fourth year. One of the blind ones has died within the last few years. He is of the opinion that all can be benefited by an operation, and the younger members probably cured. The oldest, now twenty-two, it having been eighteen years since the retina performed its peculiar function, could not have her vision perfectly restored. He also knows of a family in which there are four children affected with strabismus of same degree. All were operated upon, and the strabismus cured.

Dr. Miller thought one of the most incomprehensible things in medicine was the hereditary tendency given to not only certain diseases, but anatomical conformations and peculiarities of father. Noticeable and inexplicable was the almost certainty of the son beginning to become bald at the same age that his father did.

Academy adjourned.

